# Virulence Factors in *Mycobacterium tuberculosis* Infection: Structural and Functional Studies

**DOI:** 10.3390/biom13081201

**Published:** 2023-07-31

**Authors:** Rita Berisio, Alessia Ruggiero

**Affiliations:** Institute of Biostructures and Bioimaging (IBB), National Research Council (CNR), I-80131 Napoli, Italy

Tuberculosis (TB) remains one of the main causes of death by infection, especially in immunocompromised patients. High rates of TB antibiotic resistance have been observed around the world, making these bacteria immune to anti-TB drugs. Kids and adults with other disease like HIV are more prone to be affected. In most healthy people, the immune system can destroy the bacteria, whereas in some cases, TB infection begins without symptoms before becoming active (latent TB). TB can persist in a reservoir of infected individuals in this latent state for many years and can be reactivated to cause disease. Up to 10% of people with latent TB develop the disease, through a complex mechanism which is still not fully understood [1,2,3,4,5,6,7].

New treatment strategies are strongly needed to combat the global tuberculosis epidemic and the spread of drug resistant tuberculosis, and to tackle the threat of *M. tuberculosis* resuscitation. To address this issue, comprehensive knowledge of important macromolecules regulating *M. tuberculosis* physiopathology is essential. A significant outcome has been derived in the last few decades, by studies aimed to the structural and functional characterization of several key actors of TB life cycle and virulence. Many of them are involved in regulatory mechanisms, such as cell division, peptidoglycan synthesis and degradation, and host–pathogen interaction. This Special Issue offers an open access platform that aims to bring together a collection of research articles, reviews, and perspectives to address various aspects of the *M. tuberculosis* life cycle for the development of novel approaches to fight TB. 

Here, Dimitrov et al. evaluated toxicity and oxidative stress of two selected nitrofuranyl amides (DO-190 and DO-209) with high in vitro antimycobacterial activity. Acute toxicity tests showed that no changes were observed in the skin, coat, eyes, mucous membranes, secretions, and vegetative activity in mice. Moreover, the histological findings include features consistent with normal histological architecture without being associated with concomitant pathological conditions. The two compounds disturb the oxidative balance in mouse liver, but further investigations are needed to elucidate the mechanisms of possible hepatoprotection. Molecular docking analyses showed promising protein–ligand interactions for both molecules. Thus, both studied compounds displayed promising activity with low toxicity and can be considered for further evaluation and/or lead optimization [8].

Moreover, Grininger et al. discuss structural features of an important druggable target against *M. tuberculosis*, Rv0183/mtbMGL [9]. This enzyme is a monoacylglycerol lipase of *M. tuberculosis* and is involved in providing fatty acids and glycerol as building blocks and as an energy source. Since the lipase is expressed during the dormant and active phase of an infection, Rv0183/mtbMGL is an interesting target for inhibition. Authors present the crystal structures of a surface-entropy-reduced variant K74A Rv0183/mtbMGL in its free form and in a complex with a substrate-mimicking inhibitor. The two structures reveal conformational changes in the cap region that forms a major part of the substrate/inhibitor binding region. This work demonstrates the high conformational plasticity of the cap from open to closed conformations and provides useful insights into changes in the substrate-binding pocket, the target of potential small-molecule inhibitors. 

The CRISPR-Cas system is an adaptive immune system for many bacteria and archaea to defend against foreign nucleic acid invasion, and this system is conserved in the genome of *M. tuberculosis*. Wei et al. identify a transcription factor, denoted as CasR (CRISPR-associated protein repressor, encoded by Rv1776c), that binds to the upstream DNA sequence of the CRISPR-Cas gene cluster both in vivo and in vitro, and regulates the expression of cas genes [10]. A panoramic of emerging pharmacological strategies that target major virulence factors of antibiotic-resistant *M. tuberculosis* is reported by Italia et al [11]. Authors present a comprehensive overview of drugs and drug candidates that target cell walls, envelopes, and secretory systems. Information at the molecular level of *M. tuberculosis* pathogenesis is provided, and potential future directions in therapeutic strategies are suggested to access new drugs to combat the growing global threat of antibiotic-resistant *M. tuberculosis* infection. 

A World Health Organization (WHO) initiative, The End Tuberculosis Strategy, set ambitious targets for 2020–2035, a 90% reduction in TB incidence and 95% reduction in TB deaths by 2035, compared with 2015 and, beyond this, the elimination of the disease as a global health problem by 2050. To achieve these goals, both drug and vaccine development are needed. Currently, BCG is the only vaccine approved to prevent TB. Most evidence shows that BCG confers protection against severe forms of childhood TB, but in adults and those with comorbid conditions its effect could not prevent the disease at all. Other strategies include recombinant BCG (rBCG) to improve BCG’s efficacy and to use as an alternative to BCG in vulnerable populations. Several studies have shown that *M. tuberculosis* culture filtrates contain proteins that have promising vaccine potential. Here, Choi et al. describe the potential of Rv1876 bacterioferritin, identified from the culture filtrate fraction, with strong immunoreactivity [12]. Its immunobiological potential has not been reported previously. They show that recombinant Rv1876 protein induces dendritic cell (DCs) maturation by MAPK and NF-kB signaling activation. Also, it induces a T helper type 1 cell-immune response and expands the population of the effector/memory T cell. Boosting BCG with Rv1876 protein enhances the BCG-primed Th1 immune response and reduces the bacterial load in the lung, compared to those of BCG alone. Thus, Rv1876 is a good target for the prime-boost strategy. These authors have previously shown the successful conjugation of several subunit antigens with Early Secretory Antigenic Target-6 (ESAT-6) in eliciting an immune response [13,14,15,16]. Besides its interest as a vaccine antigen, ESAT-6 plays a central role in several adaptation mechanisms of *M. tuberculosis* to the host. Anes et al. provide a narrative review that highlights the recent advances in understanding the role of ESAT-6 in hijacking macrophage function to establish successful infection and transmission, and also, its targeting for the development of better diagnostic tools and future vaccines for human TB [17].
